# Effectiveness of surveillance technology for the prevention of suicides in public spaces: a systematic review

**DOI:** 10.1136/bmjph-2025-004286

**Published:** 2026-07-01

**Authors:** Laura Joyner, Bethany Cliffe, Jay-Marie Mackenzie, Keith Hawton, Peter Craig, Lisa Marzano

**Affiliations:** 1 Department of Psychology, Middlesex University, London, UK; 2 Department of Psychology, University of Westminster, London, UK; 3 Department of Psychiatry, Centre for Suicide Research, University of Oxford, Oxford, UK; 4 School of Health and Wellbeing, University of Glasgow, Glasgow, UK

**Keywords:** Systematic Review, Public Health, Public Health Practice

## Abstract

**Objective:**

The use of surveillance technologies (eg, security cameras, motion sensors, video analytics) has been recommended for supporting suicide prevention efforts in public spaces. We sought to identify and synthesise the current evidence on the impact of surveillance technologies deployed in such environments on suicide and related outcomes.

**Design:**

Systematic review without meta-analysis.

**Data sources:**

We conducted systematic searches between 1990 and August 2025 on the following databases: PsycINFO, MEDLINE, CINAHL, Computer Source, Ovid, SPP, Web of Science, PTSDpubs, CENTRAL, ACM DL and IEEE Explore. Our searches also extended to grey literature from relevant websites.

**Eligibility criteria:**

Studies were included if they assessed the deployment of surveillance technology at a public location on suicide-related outcomes (including suicides, suicide attempts, rescue interventions and trespass events).

**Data extraction and synthesis:**

Two reviewers independently screened studies and extracted data. Study results were synthesised using narrative and tables, and the quality of studies was assessed using the Mixed Methods Appraisal Tool.

**Results:**

The searches identified 3039 items, with 15 studies meeting the eligibility criteria. Just one study provided clear evidence of a reduction in suicides (IRR=0.37, 95% CI 0.26 to 0.54) following the installation of a ‘smart’ surveillance system on a bridge, but its use of tension-wire sensors also restricted ease of physical access. The presence of closed-circuit television cameras was associated with a 7% reduction in rates of suicide at local rail stations in one study (IRR=0.93, 95% CI 0.88 to 0.98), but the presence of cameras was not a significant factor in two metro station studies. Additionally, two studies observed an increase in interventions (eg, rescue responses) following the installation of smart surveillance technologies on bridges (140% and 520% increases, respectively), but saw no change in suicide rates. Three studies indicated that smart surveillance technologies with audible deterrents may reduce trespass to dangerous sites, but their relevance to suicide prevention is unclear.

**Conclusions:**

The current evidence base to support the use of surveillance technologies for preventing suicides is limited. There is a clear need for further evaluations of surveillance-based interventions, including their implementation, associated measures and impacts to inform development of effective suicide prevention initiatives.

**WHAT IS ALREADY KNOWN ON THIS TOPIC**

**WHAT THIS STUDY ADDS**

- Our systematic review found 15 studies that examined the effects of installing surveillance technologies on suicides or trespassing at public locations, but overall, the evidence supporting their effectiveness for suicide prevention was limited.
- At some locations, the installation of surveillance technologies was associated with an increase in numbers of interventions or police call outs, but not a decrease in deaths.

**HOW THIS STUDY MIGHT AFFECT RESEARCH, PRACTICE OR POLICY:**

- The findings highlight critical gaps in the current evidence, particularly in regard to the use of ‘smart’ or ‘intelligent’ surveillance technologies.
- Further research is also needed to understand the impact of processes surrounding surveillance technologies on their effectiveness (eg, rescue response times).

## Introduction

 Approximately 7000 suicides are recorded annually in the UK,^1^ with around one-third of these deaths occurring in public locations, such as railways, cliffs, roads and bridges.^2^ These deaths can have a lasting impact, not only on family and friends, but also for potential witnesses and staff dealing with their aftermath. There can also be substantial financial implications for affected sectors. For example, each suicide on the railways in Great Britain is estimated to cost the industry approximately £275 000,^3^ due in part to factors related to service disruptions (eg, delay penalties) and staffing (eg, replacement crews, driver leave).

Strategies to prevent suicides in public locations are often categorised into four general approaches: (1) restricting access to the site and means of suicide, (2) increasing opportunity and capacity for human intervention, (3) enabling help-seeking and (4) changing the public image of the site.^4^ While measures that fully restrict access to means (eg, installing barriers over 2.3 metres) have proven effective in preventing suicides in some environments,^5 6^ such approaches are not always practical or feasible to instal everywhere (eg, due to engineering constraints). Another ‘hard’ measure (ie, a physical, mechanical or technological intervention) recommended in national guidance is the introduction of surveillance technologies.^4^

Previously, authors of systematic reviews of suicide prevention measures at high-risk locations have discussed surveillance cameras in the context of ‘increasing opportunity for human intervention’.^7^,^9^ However, others have noted that such systems require ongoing monitoring by vigilant staff to identify potential suicide attempts in real-time^10^ and therefore their use may not always be practical. However, by integrating alternative data sources (eg, heat sensors) and/or tools (eg, analytics to detect motion in a scene) ‘Smart’ Surveillance Technologies (SST) can automate aspects of the surveillance process in ways that may alleviate some (or all) of the monitoring burden for staff.^11^ In some cases, they may also be designed to issue a fully automated response (eg, audible deterrents, responsive lighting). SSTs have been proposed, recommended and used in suicide prevention efforts for some time,^12 13^ and are increasingly becoming more complex, technologically advanced, and commercially available. Examination of if, and when surveillance technologies (including SSTs) may help prevent suicides in public locations is warranted in order to inform future suicide prevention initiatives. The aim of this paper is to examine the existing literature on the effects of surveillance technology on suicide-related outcomes within public spaces.

## Method

The review protocol was registered (PROSPERO ID CRD42024495308) on 17 January 2024. This work was part of a wider review looking at the use of surveillance in suicide prevention at public locations. However, for clarity, other objectives from this review (eg, feasibility) will be explored elsewhere.

### Eligibility criteria

For the present work, key outcomes included: (1) deaths by suicide, (2) suicide attempts and (3) intervention events (callouts, human intervention, calls to telephone hotlines, etc). In line with previous academic research,^14^,^17^ outcomes related to trespass were also included as an associated and preventable high-risk event (for the objective of the present review only). This is in part because a degree of trespass may also be required to access means for suicide at some high-risk locations (eg, if accessing trackside areas on the railway). Surveillance technology deployed at such sites therefore has the potential to help prevent suicides by spotting unauthorised persons situated in restricted areas but may be unable to distinguish these individuals from those trespassing for other reasons.^18^ Approaching trespass and suicide prevention in an integrated manner is seen by some industries as being beneficial,^19^ with surveillance technologies having been identified by national and international industry organisations as mitigations suitable for both.^20 21^ Additionally, only studies with examples of surveillance technologies being used to monitor behaviour within public spaces (eg, railways, bridges, coastal locations) were included in the present review.

Synthesis was initially grouped into themes according to the research questions and type of automated detection employed by the system. These were ‘no smart monitoring’ (eg, CCTV), ‘detect presence’ (eg, motion sensors), ‘detect specific action’ (eg, linger detection), ‘detect emotional states’, ‘identify specific people’ and ‘forecasting risk of attempt’. Coding of these modalities was based on the behaviours and/or characteristics the SST sought to detect and may therefore differ from formal technological or author descriptions. Where a single study employed more than one modality, categorisation was prioritised based on whichever was the more advanced category. For instance, an intervention employing both intrusion detection and behavioural detection would have been categorised under the latter.

### Identification of eligible studies

The following databases were searched: PsycINFO, MEDLINE, CINAHL, Computer Science, Ovid, SPP, Web of Science, PTSDpubs, CENTRAL, ACM DL and IEEE Explore. Searches also extended to grey literature including government, business, industry and third-sector reports and non-peer reviewed academic work. The full search strategy can be found in online supplemental appendix A. An initial search was run in March 2024 with follow-up searches in October 2024 and August 2025 to ensure publications in this base review were up to date. Any updates from subsequent searches during the course of the project will be published in a public OSF project (https://osf.io/jvmxf/overview?view_only=d0636100198b44cc9328495025ce94a9). No restrictions were applied regarding publication type. Only studies published in English since 1990 were included.

After the initial round of searches was completed, files containing search results were uploaded onto Covidence for deduplication. Two researchers (LJ and BC) independently screened titles and abstracts on Covidence and crosschecked for agreement. The same reviewers then independently screened the remaining full-text articles, documenting reasons for exclusion at this stage. Disagreements were discussed and resolved between the two reviewers.

### Data collection and quality assessments

A data extraction tool developed using Microsoft Excel was piloted prior to data capture. Two researchers (LJ and BC) extracted data from papers and cross-checked 10% of extracted data to ensure accuracy. Any disagreements unresolvable by discussion were referred to a third reviewer (J-MM or LM). An overview of extracted data can be found in online supplemental appendix B. Quality appraisals were carried out using the Mixed Methods Appraisal Tool (MMAT).^22^ Studies were first checked for eligibility using the MMAT’s two screening questions, and eligible studies were rated against five criteria from the Quantitative non-randomised category. Ratings of ‘yes’ were assigned where studies reported information demonstrating they had met the criterion, otherwise they were given ratings of ‘no’ or ‘can’t tell’. The quality appraisals were conducted by one reviewer (LJ) and checked for accuracy and consistency by a second (BC), with discrepancies resolved by discussion (online supplemental appendix C).

### Data synthesis

Extracted data were synthesised using narrative and tables. Meta-analysis was not conducted as data from the relevant studies were not sufficiently homogeneous.

### Patient and public involvement

People with lived experience of suicide have been involved at several key stages of our project. Lived Experience Experts from the National Suicide Prevention Alliance were consulted prior to making the funding application associated with this work, and provided feedback on the project’s aims and objectives, methods and dissemination plans. During the course of the project, we have continued to meet regularly with our Lived Experience Advisory Group (LEAG), whose feedback has helped us to make sense of some of the potential risks surrounding the different technological responses, for example. A member of the LEAG also sits on our project steering group where, as a direct result of their feedback, lived experience feedback and consultation are included as a prominent agenda item.

## Results

**Figure 1:**
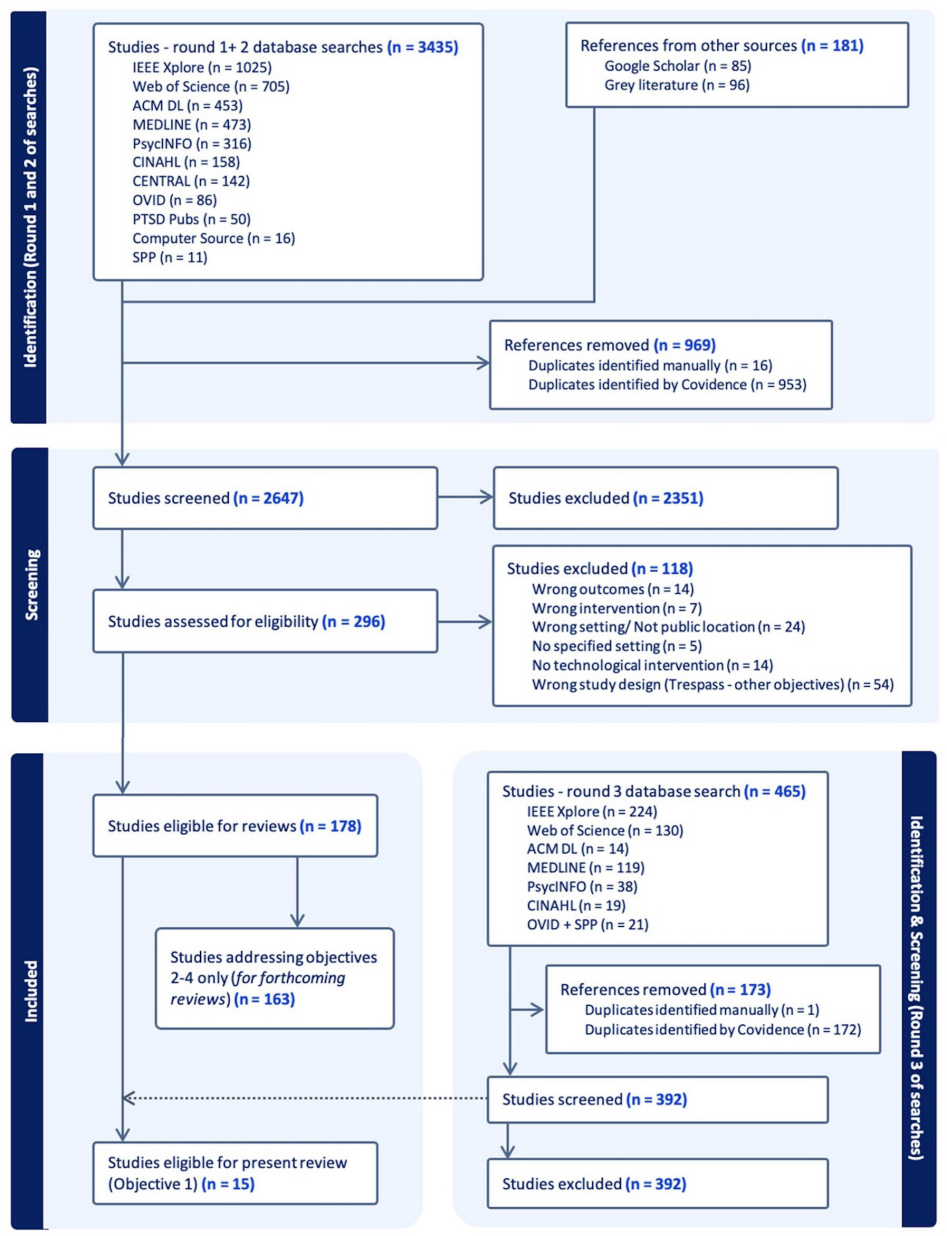
Preferred Reporting Items for Systematic Reviews and Meta-Analysis (PRISMA) flow chart.

After removal of duplicates, a total of 2647 items were identified by 16 October 2025. After initial screening of titles and abstracts, 296 items were assessed for eligibility. Of those, 118 were excluded at this stage, with reasons for exclusion provided in figure 1. A total of 178 documents included discussion of the role of surveillance technologies in suicide prevention. However, only 15 of these fulfilled the criteria for inclusion in the present work, that is, looked at effectiveness (to ensure clarity, the remaining papers are examined separately in a forthcoming scoping review).

Follow-up searches for the present review were carried out between 21 August 2025 and 27 August 2025, where a further 392 items were identified after removal of duplicates. However, none of these additional items identified in this follow-up search fulfilled the criteria for inclusion in the present review.

### Study characteristics

The included studies came from eight countries: Australia (n=5^23^,^27^), South Korea (n=4^28^,^31^), Austria (n=1^32^), Belgium (n=1^33^), Denmark (n=1^34^), Finland (n=1^35^), Sweden (n=1^36^) and the USA (n=1^37^). Three of these studies examined the same project implemented at a coastal location in Australia, each at a different point in time.^24^,^26^ A further five studies focused on bridge locations,^2328^,^31^ with potentially up to three having investigated different interventions deployed on the same bridge in South Korea.^28 29 31^ Another seven studies focused on railway or metro locations, and, of these, four were of suicide-related outcomes at stations and/or their surrounding environments.^27 32 34 36^ The remaining three rail (trespass-focused) studies examined systems installed at a rail bridge,^37^ a tunnel entrance^33^ or at lineside locations.^35^

11 of the 15 studies included deaths by suicide as an outcome, with four also analysing suicide attempts; however, this outcome was not clearly defined across all studies. Single studies investigated intervened suicidal acts,^30^ police call outs and successful sightings on closed-circuit television (CCTV)^24^ and number of rescue attempts.^29^ Three studies had trespass events as the main outcome. Of these, one formed part of a technical report^37^ and a second was included in a presentation of an industry-led trial.^33^

### Characteristics of the interventions

**Figure 2:**
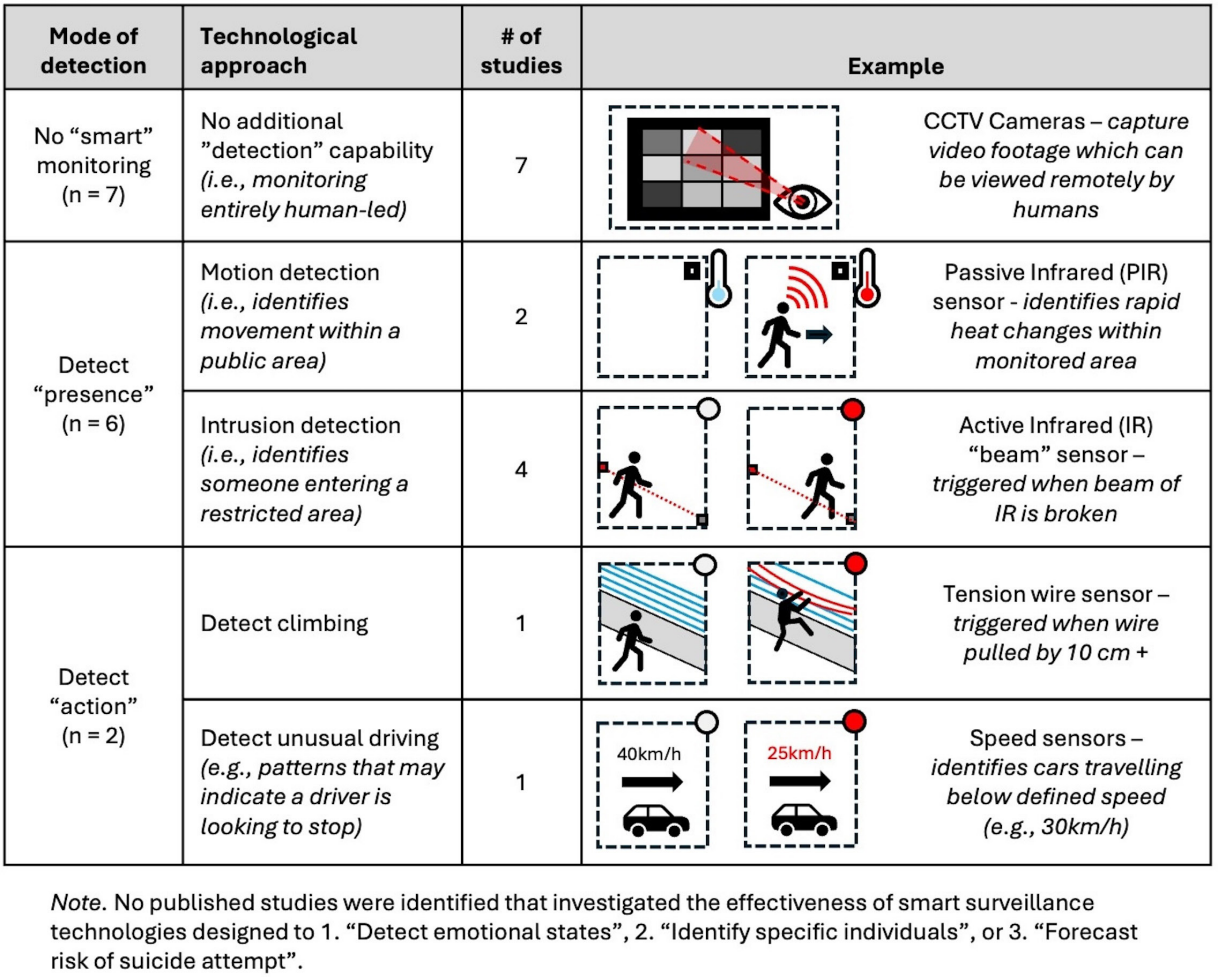
Examples of identified systems by mode of detection. CCTV, closed-circuit television.

Nearly half of the studies (7/15) included the use of systems that we primarily categorised as ‘not smart’, for example, CCTV.^23^,^2732 36^ These included one study^32^ where the CCTV was monitored by security officers at the locations as part of their role (eg, rather than the system playing a role in identification, see figure 2). Another CCTV system examined across three studies^24^,^26^ was initially monitored by a security company on police request only (however, ‘smart’ upgrades during the postintervention period for two of these studies meant the system later issued automated alerts when barriers were climbed). In the three other studies, it was unclear whether the CCTV systems were or could be monitored in real-time.^23 27 36^

A further six studies focused on interventions which included the use of SSTs designed to ‘detect the presence’ of an individual within or entering an area of interest.^2829 33^,^35 37^ The remaining two studies examined interventions which included SSTs that we classed as detecting a specific ‘action’ (eg, vehicle slowing,^30^ climbing^31^). To date, we have identified no studies which have investigated the effectiveness of any SSTs designed to ‘detect emotional states’, ‘identify specific individuals’, or ‘forecast risk of attempt’ (eg, based on exhibited behaviours) at reducing suicide or trespass-related outcomes.

**Figure 3:**
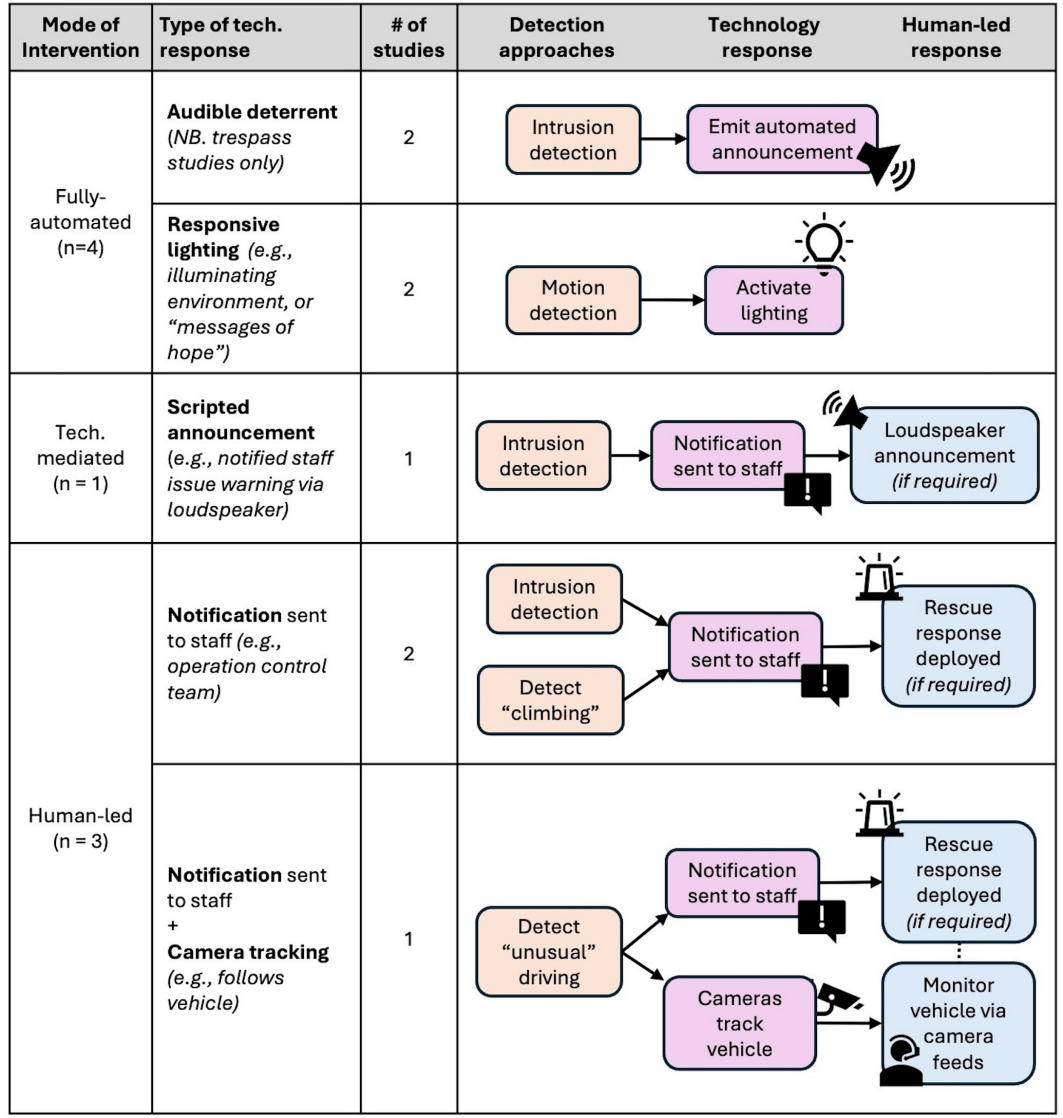
Examples of identified ‘smart’ systems by mode of response.

Across the eight ‘SST’ studies, four^2833^,^35^ included technology designed to operate as stand-alone interventions (ie, fully automated, see figure 3), two of which were tested in relation to trespass outcomes only.^33 35^ Another study tested an SST which notified staff who were then to use the system’s speakers to issue a scripted trespass intervention, that is, technology-mediated intervention.^37^ The remaining three studies focused primarily on SSTs designed to notify staff who would then initiate a rescue response, that is, human-led intervention.^29^,^31^

Summaries of individual studies can be found in table 1.

**Table 1:** Summary of studies

Study	Tech. focus of paper?	Type of SST	Duration of data	Study design	Other interventions present?	Outcome of interest	Conclusions	Comments/limitations
Australia: Bridge over water								
Kõlves et al., 2023 (Academic Research Letter: Peer reviewed)	Partly	Mode: Not smart Input: Surveillance Camera Response: Not specified	20 years (2001–2021)	Pre–Post, that is, before and after installations (Cameras and phones installed in 2012. Barriers installed in 2015.)	Yes. Lifeline phones installed at the same time as cameras. Barriers (3 m*) installed at later date during study.	Suicide	Evidence of an initial decline in suicides at the bridge from 2013 (the year after cameras and phones were installed) until 2021 (APC=−31.6%, 95% CI −44.9% to −15.1%, p=0.002). Fine-grained analysis identified 2012 as when deaths began to decline at the bridge. The barrier installation in 2015 was then associated with a greater reduction in deaths, declining close to zero.	Initial analysis grouped data into 3-year blocks to avoid small numbers, and as such any reduction was only apparent in fine-grained analysis. Not possible to separate any effect of cameras from phone installation. Data between 2019 and 2021 from police reports only as coronial findings were not available at the time.
Australia: Cliff site								
Lockley et al., 2014 (Academic Journal Paper: Peer reviewed)	Partly	Mode: Not smart. Input: CCTV Cameras Response: Monitored by CCTV operators at security company (on police request only)	Suicide data: 11 years (2001–2011) Reported jumping incident data: 7 years (2006–2012) CCTV monitoring data: 3 years (2010–2012)	Pre–Post, that is, before and after installations (Interventions implemented in 2010–2011. CCTV installed in 2010)	Yes. Wider project included new fencing, crisis telephones, signage and landscaping works.	Jumping incidents Suicide Police call outs Successful sighting on CCTV	No significant changes to trends for jumping incidents and deaths by suicides were observed across the time period. There was some initial suggestion of a decrease in reported jumping incidents over time; however, this trend was not significant (EAPC = −2.61%, 95% CI −21.1% to 20.2%, p=0.76). The trend for the number of deaths by suicide was also not found to be statistically significant (EAPC=6.71%, 95% CI −2.5% to 16.8%, p=0.14). Significant increase in police call-outs (EAPC=12.89%, 95% CI 0.3% to 27.1%, p<0.05); however, this rise began before the interventions were implemented and was primarily driven by an increase in the number of call-outs relating to individuals approaching or located at the site (EAPC=16.04%, 95% CI 7.1% to 25.7%); p<0.001). Relative to all call outs, the proportion of individuals who had crossed the fence appeared to decrease over time (EAPC=−13.77%, 95% CI −25.0% to −0.9%, p<0.05). For years active, the number of CCTV monitoring requests from police increased over time (47 in 2010 to 131 in 2011, and 203 in 2012). The proportion of requests with successful sightings also increased over this time (9% in 2010 and 10% in 2011, to 40% in 2012).	The authors suggest one reason the proportion of ‘over the fence’ call outs decreased may in part be due to CCTV aiding earlier interventions. However, improvements to the fencing are also thought to have played a key role. Increases in the proportion of successful sightings were attributed by stakeholders to improvements in the camera technology. Authors note stakeholders felt the cameras were a key factor in improvements in police responsiveness and that cameras helped to speed up the search process generally. Cannot make simple pre–post comparisons due to multiple measures and camera upgrades. Relatively limited time had passed since measures were implemented in terms of the jumping incident and suicide death data.
Ross *et al*., 2020 (Academic Journal Paper: Peer reviewed)^25^	Partly	Initial mode: Not smart Input: CCTV Camera response: Monitored by CCTV operators at security company (NB. ‘smart’ additions also reported at the site in 2013: Barrier-mounted sensors to detect climbing; alerted security monitoring company)	17 years (2000–2016)	Pre–Post, that is, before and after installations (Primary interventions implemented in 2010–2011. CCTV installed in 2010)	Yes. Wider project also included crisis telephones, signage and protocols with police.	Suicide	Joinpoint analysis suggested there was a slight increase in deaths across the data collection period (2000–2016); however, this trend was not significant (APC=5.41%, 95% CI −0.38 to 11.53, p=0.07). Analysis by sex showed an upward trend in suicide in males across the same period (APC=6.23%, 95% CI −0.31 to 130.30, p=0.06). However, the Joinpoint analysis showed a significant change to trends in suicide in females. Between 2000 and 2010 an upward trend in suicide in females was observed (APC=16.64%, 95% CI 8.18 to 25.76, p<0.001). This was then followed by a downward trend in 2010–2016 (APC = −21.27%, 95% CI −33.14 to −7.30, p<0.01).	A significant reduction in female suicides coincided with the implementation of a package of measures at this site. Qualitative feedback from police in this mixed-methods study suggests that the CCTV and alarms were indeed useful for locating and detecting attempters. However, the authors also indicated that the fencing may have proven more of a psychological deterrent for women. Not possible to separate any effect of implementation from other measures. Lack of power to detect statistically significant changes. Issues obtaining accurate suicide attempt data for the site.
Torok *et al*., 2023 (Academic Journal Paper: Peer reviewed)^26^	Partly	Initial mode: Not smart Input: CCTV Camera response: Monitored by CCTV operators at security company (NB. ‘smart’ additions also reported at site in 2013: Barrier-mounted sensors to detect climbing; alerted security monitoring company)	14 years (2006–2019)	Pre–Post, that is, before and after installations Pre: 2006–2011 Post: 2012–2019 (NB. Further CCTV cameras also added during ‘post’ stage)	Yes. Wider project included new fencing, crisis telephones, signage and landscaping works). Also, virtual fences commissioned in 2013 (identified by authors but not included in analysis)	Suicide (by jumping) NB. as well as the ‘immediate’ area, data was also collected for ‘local’ (3 km both directions along coastline), and ‘broader’ areas (18 km both directions along coastline)	Joinpoint analysis indicated that between 2006 and 2019, there was a slight downward trend in suicides within the immediate area, but this trend was not significant (APC = −1.95%, 95% CI −6.9 to 3.3, p=0.14). χ^2^ tests showed there were also fewer suicides than expected in the immediate area after project implementation, but any difference was not statistically significant (χ^2^(1) =1.10, p=0.18). Some evidence of displacement across identified ‘hotspots’ within the immediate area. Significantly greater number of actual (vs expected) deaths at ‘lookout point’ post installation (z=1.80, p=0.05). However, while not statistically significant, there were fewer actual (vs expected) deaths at a second hotspot only 500 m away, within the immediate project area (z=1.60, p=0.08). No significant overall difference in distribution of suicides before and after project implementation and across the geographic areas.	Authors note that immediate displacement findings may suggest individuals could be willing to seek out alternative spots in an immediate area that are less visible or provide fewer impediments to access. Only deaths where location could be identified with 500 m precision were included in the analysis. Not possible to separate any effect of implementation from other measures, including addition of virtual fence during the ‘post’ period.
Austria: Subway stations								
Niederkrotentha-ler et al., 2012 (Academic Journal Paper: Peer reviewed)	No	Mode: Not smart. Input: Video Surveillance System Response: Monitored by security officers	31 years (1979–2009) (NB. Stations varied in the length of time they were open, but the end point was December 2009 for all)	Cross-sectional observational study Structural, contextual and passenger characteristics of 84 subway stations entered into Poisson regression models to account for differences in fixed characteristics of stations and changes over time (one included characteristic was ‘Surveillance units’ present at 16 stations on opening)	Yes. Security officers for sites with the ‘surveillance units’ installed. Whole subway network approach, so other interventions included in the analysis.	Suicide Suicide attempt	After full adjustments in the model for other environmental factors (eg, train speed), there was no significant association between the presence of surveillance and rates of deaths by suicide (RR=1.44, 95% CI 0.90 to 2.27) or suicide attempts (RR=1.15, 95% CI 0.65 to 2.01).	Prior to controlling for other factors, presence of surveillance units was positively and significantly associated with suicide deaths and attempts. However, units were also not implemented for suicide prevention specifically. Limited statistical power means small effects may not be detected. Ecological design means causality cannot be inferred.
Australia: Railways								
Too *et al*., 2015 (Academic Journal Paper: Peer reviewed)^27^	No	Mode: Not smart. Input: Video Surveillance System (analysis based on number of ‘surveillance units’) Response: Not specified	12 years (2001–2012)	Cross-sectional observational study Social, economic and physical factors of neighbourhoods entered into negative binomial regression models to identify factors associated with rail suicide risk. (One included characteristic was the number of ‘surveillance units’ at stations and station car parks within the postcode)	Not specified. NB. Other factors added to model included rail-related factors (eg, train speed) and factors relating to local environment	Suicide	After accounting for other environmental and person-level factors, increased numbers of surveillance units at stations/car parks in a neighbourhood were associated with reduced risk of railway suicides (IRR=0.93, 95% CI 0.88 to 0.98).	Prior to accounting for other factors, number of surveillance units was positively associated with suicide risk (IRR=1.04, 95% CI 1.01 to 1.07). Authors suggest this may be due to correlation between station density and number of surveillance units. Authors suggest actual number of suicides may have been underreported in the data due to coronial delays or misclassification of cause of death.
Sweden: Subway stations								
Uittenbogaard & Ceccato, 2015 (Academic Journal Paper: Peer reviewed)^36^	No	Mode: Not smart. Input: CCTV Cameras (present and easily visible) Response: Not specified	14 years (2000–2013) 22 stations: ‘City centre’ 78 stations: ‘Periphery’ (of city)	Cross-sectional observational study Environmental and socioeconomic characteristics of 100 subway stations entered into ordinary least squares regression model to identify factors associated with suicide rates. (One included characteristic was the presence of visibly placed CCTV cameras)	Not specified. NB. Other factors added to model relate to station characteristics (eg, main station, underground), and to the local environment (eg, population density, young male population)	Suicide	Presence of CCTV was not a significant predictor of suicide rates across any of the tested models.	Authors noted that the rarity of suicide events mean the results should be interpreted with caution. Changes in camera numbers/placement at any site across the time period may not be accounted for in the audit data (ie, was collected at fixed point)
Denmark: Railway Station								
Erlangsen *et al*., 2023 (Academic Journal Paper Short Report: Peer reviewed)^34^	*Partly*	Mode: Detect presence (Motion) Input: ‘Motion detection’ Response: Motion-sensitive lights	‘Previous incident review’ data: 2012–2018 Post installation data: 16 months (January 2020–April 2021)	Pre–post, that is, before and after installation (descriptive statistics only) (Lighting, signs and barriers installed December 2019)	Yes—12 signs encouraging help-seeking and physical barriers at platform ends added at same time.	Suicide Suicide attempt (Primary study focus—calls to suicide prevention helpline)	In the 16 months after implementing the measures, no suicide deaths had occurred, and only one suicide attempt. Prior to this there had been an average of 1.5 deaths per year.	The authors suggested that the motion-sensitive lighting may have limited the appeal of dark spots in the station. However, short timeframe means it was not possible to produce conclusive evidence. Not possible to distinguish the influence of motion-sensitive lighting from the physical measure and help-seeking signs. Only tested at one station. Paper only presents descriptive statistics
USA: Railway bridge over water								
da Silva *et al*., 2006 (Project Report: No peer review)^37^	Yes	Mode: Detect presence (intrusion) Input: Dual-technology motion detector (stereo doppler microwave and passive infrared sensor), Magnetometer. Video camera and infrared illuminator Response: Notification–security company control room. Loudspeaker for scripted announcement	3 years (August 2001–August 2004) (NB. Trespass event data based on 129 matched days per year where system was operational)	Observational study (descriptive statistics only) Trespass data reported annually following system installation	No	Trespass event	There was a decrease in trespass events of 60% from 46 in the active days for year 1 vs 18 in year 2. However, there was an increase to 38 trespass events in year 3. This reflects a 17% reduction from year 1.	The authors note that many trespass events were triggered by those who repeatedly returned. They suggest these individuals may have become desensitised to the system over time, as police would not be called unless they remained at the site. During the second year of the trial, there was increased media coverage of the pilot due to the marking of 5 years since the fatalities of two teenagers at the location. As such, the decrease observed in year 2 may also be attributable to media effects.
Finland: Railway lineside								
Kallberg & Silla, 2017 (Academic Journal Paper: Peer reviewed)^35^	Yes	Mode: Detect presence (Intrusion) Input: Infrared ‘beam’ sensor Response: Audible deterrent—prerecorded message.	Site A (2013): Pre=47 days, Post=67 Days Site B (2013): Pre=15 days, Post=54 days	Pre–post, that is, before and after installation (Tested across two sites)	A camera with inbuilt motion detection was also used at site to collect evaluation data.	Trespass event	Average daily trespasser count reduced by 44% at Site A (λ=0.56, 95% CI 0.50 to 0.62) Average daily trespasser count reduced by 18% at Site B (λ=0.82, 95% CI 0.70 to 0.94)	Authors note any effect may have depended on perceived risk of accident or punishment. Also, that any fears of punishment may diminish over time if not actioned. Relatively short trials. As no control sites included, authors note that other factors such as weather, daylight hours, etc are likely to have influenced effectiveness.
Belgium: Railway tunnel next to station								
Van Overmeiren, 2019 (Presentation of technical report: No peer review)^33^	Yes	Mode: Detect presence (Intrusion) Input: CCTV Camera (with intrusion detection algorithm), IR sensor Response: Audible deterrent	4 years (2016–2019)	Pre–post, that is, before and after installation (descriptive statistics only) Pre: 2016–2017 Post: 2018–2019	No	Trespass event	There was an 80% decrease in trespass events from 11 before the technology was implemented to 2 in 2018. There were 0 trespass events identified in 2019.	The installation was also associated with a 14.8% reduction in delay minutes in the section between 2017 and 2018 (800 min reduction).
South Korea: Bridge over water								
Kim *et al*., 2019 (Academic Journal Paper: Peer reviewed)^28^	Partly (Paper discusses various case studies from South Korea)	Mode: Detect presence (Motion) Input: ‘Sensors’ placed along bridge guardrails Response: Dynamic lighting illuminating ‘messages offering hope or humour’	4 years (2011–2014)	Observational study (descriptive statistics only) Rates on bridge with intervention (I) compared with rates across five other bridges (A–E) in same city. (NB. Other sources suggest intervention installed September 2012)	None identified by authors. However, dates crossover with installation of system from Lee *et al*.^29^	Suicide Suicide Attempt	The number of deaths at the bridge (I) remained relatively stable across the study period (around 5–6 per year). However, across the five other bridges in the city (A–E) there was a reduction in deaths over time (average year 1: 10.2 deaths, average in both year 3 and 4: 0.6 deaths). However, the number of attempts increased year on year at bridge I from 11 in 2011 to 184 in 2014, an almost 16 times increase across the period (change of +1573%). Comparably, across bridges A–E the number of attempts almost doubled from an annual average of 20.0 to 38.8 across the same time period (change of +94%).	Authors suggest that the increase in attempts at both Bridge I and Bridges A–E may in part be due to the substantial media attention the project attracted. Number of actual deaths at the bridge did not increase. Suggested this may be due to increased focus from relevant government departments/other organisations that helped facilitate effective interventions. Not possible to distinguish the influence of alternative factors (eg, media effects, adapted intervention processes in the city) during this time. Paper only presents descriptive statistics. No definition of ‘suicide attempt’ provided. However, numbers correspond with those reported in Lee *et al*^29^ and therefore may possibly reflect both ‘rescues’ on bridge and water.
Lee *et al*., 2016 (Academic Journal Paper: Peer reviewed)^29^	Yes	Mode: Detect presence (Intrusion) Input: Active infrared sensor (attached to top of railings). Also, smart camera (analytics: eg, intrusion detection zone) Response: Notifications sent to staff.	Bridge A: 2 years (2012–2013) Bridge B: 1 year (‘post’ period only–2013)	Pre–Post, that is, before and after installations Pre: 1 January 2012–31 December 2012 Post: 1 January 2013–31 December 2013	Yes. Emergency call buttons. Dates may also crossover with installation of system from Kim *et al*.^28^	Rescue on bridge Rescue on water	At bridge A, rescues increased from 15 in the year before installation to 93 in the trial year (520% increase). Of rescues in the trial year, 91.3% occurred on the bridge (vs on water). At bridge B, eight rescues occurred on the bridge during the trial year (no data provided for 2012).	Placement of sensors on bridge barriers may help draw rescuers’ attention to suicide attempts. Impact of system on suicide deaths from the bridges unknown. After trial, operational improvements were made to the system to reduce blind spots and improve the sensor capabilities. Unclear whether any attempts were missed prior to this (ie, during ‘post’ period). Longer-term impact on rescues unknown. Paper only presents descriptive statistics.
Shin, Pirkis, Clapperton, *et al*., 2024† (Academic Journal Paper: Peer reviewed)^31^	No NB. Part of wider study on four bridges	Mode: Detect ‘action’ (climbing) Input: Tension wire sensor Response: Notifications sent to rescue team.	8 years (2013–2020)	Pre–Post, that is, before and after installations Pre: 2013–2016 Post: 2017–2020	Yes The five tension wires were part of a 1 m upper fence with spinning rail at top added to existing fence (total of 2.5 m high partially restricted barrier). Site already had CCTV, crisis phones and signage.	Suicide	Suicide rates were significantly lower in the postintervention period compared with the preintervention period (IRR=0.37, 95% CI 0.26 to 0.54). Authors note that the intervention could be more effective if spacing between the wires did not allow for humans to get through them.	Overall focus of paper on effectiveness of partial restriction of access to means. Unlike ‘virtual fences’, tension wire sensors also add a physical element to the environment. Authors note these sensors may be best thought of as temporary solutions. Not possible to separate effect of SST from other aspects of the intervention (eg, spinning rails). Also not possible to separate the sensor’s effectiveness as a surveillance vs physical measure.
South Korea: Road bridge over water								
Shin, Pirkis, Spittal, *et al*., 2025 (Academic Journal Paper: Peer reviewed)^30^	Partial	Mode: Detect ‘action’ (vehicle speeds below 30 km/h) Input: Video-based incident detection system (VIDS), Speed sensors Response: Notification sent to operation control team; Camera tracking	14 years, 1 month (2008–2022)	Pre–post, that is, before and after installations Period 1: 1 July 2008–31 December 2014 Period 2: 1 January 2015–30 November 2017 Period 3: 1 December 2017–31 July 2022	Yes. Period 1 had existing CCTV (general use) and 1 m railing initially. Period 2 VIDS and speed sensors added. Period 3 saw addition of 2 m high spinning bar rail (+ VIDS)	Suicide Intervened suicidal act (prejumping) Non-fatal suicide attempt (by jumping)	Addition of VIDS (period 2) saw a significant rise in interventions (IRR=2.40, 95% CI 1.65 to 3.78), but no impact on deaths (IRR=1.23, 95% CI 0.59 to 2.56). The further addition of spinning rails (period 3) saw an overall reduction in suicide deaths compared with both period 1 (IRR=0.28*, 95% CI 0.10 to 0.82) and period 2 (IRR=0.23*, 95% CI 0.07 to 0.71). Period 3 also saw decrease in interventions compared with the VIDS-only period (period 2 vs 3, IRR=0.37*, 95% CI 0.25 to 0.57).	Authors note VIDS may have helped to detect a wider range of crisis behaviours than previous. Suggested the increase in interventions observed in period 2 could have prevented an overall increase in deaths (not possible to determine here). Suggested VIDS may not be recognisable by individuals as a suicide prevention measure. Data supplied by bridge company, so authors were unable to evaluate for displacement, or account for co-founders.

* Height of barrier obtained from media reports.

† While the exact location of the bridge is not identified in Shin *et al,*^31^ it does appear to be the same location as studied by Lee *et al*^29^ and Kim *et al*.^28^

### Studies with systems without ‘smart’ monitoring

We identified seven studies where surveillance cameras were included in natural experiments (ie, where researchers did not manipulate exposure to the ‘intervention’^38^). Of these, three studies included the presence of ‘video surveillance units’ as a station characteristic factor in models predicting suicide rates across railway or subway networks. Niederkrotenthaler *et al*^32^ examined the influence of different station characteristics on suicide attempts and deaths between 1979 and 2009. This included the presence of surveillance units at 19% of stations on the Vienna subway network (16/84 stations), consisting of a video surveillance system monitored by officers (who also conducted patrols) installed for general use when the station opened. Prior to adjustment, Poisson regression models suggest the presence of surveillance units was one factor positively associated with rates of suicide deaths and suicide attempts. However, after adjusting for covariates such as speed of train and passenger numbers, the presence of surveillance units was no longer significantly associated with rates of suicide deaths or suicide attempts.

Similarly, Uittenbogaard and Ceccato^36^ included several station environment characteristics in their analysis of suicide deaths between 2000 and 2013 on the subway system in Stockholm, including the visible presence of CCTV. Ordinary least squares regression models showed no significant association with the presence of CCTV on suicide rates at stations across the total subway system or at 78 periphery stations. Due to multicollinearity, presence of CCTV was not included in the city centre model (22 stations). It is also unclear whether the CCTV was being monitored in real time.

Too *et al*^27^ looked at the influence of various social, economic, and physical factors on rail suicide rates across neighbourhoods in Victoria between 2001 and 2012. This included the number of video surveillance units installed at railway stations and car parks within the local area. Univariate analysis suggested a positive relationship between suicide risk and the number of surveillance units within a local area. However, in the multivariate model, the relationship became negative, which the authors suggest may be due to correlations between station density and the number of surveillance units. While it is unclear whether the system was monitored in real time, this approach may better reflect the overall (perceived) coverage of surveillance than the previous studies in station environments.

A recent study by Kõlves *et al*^23^ analysed suicide data between 2001 and 2021 from one Australian bridge location to understand the effect of different packages of suicide prevention measures installed across time. Prevention measures included the installation of crisis phones, signage, and surveillance cameras in 2012, and barriers in December 2015. To avoid small numbers, the authors also grouped data into 3-year blocks. Using Joinpoint regression analysis, they identified two changes in trends across the data collection period, including a decrease in deaths by suicide at the bridge from 2013 onwards. While descriptive statistics would indicate no change between the 3 years prior to (2010–2012) and after the installation of cameras and phones (2013–2015), the authors suggest 2012 was the start of the decline (but deemed the subsequently installed barrier to be more effective at preventing suicides). The authors also found no evidence of substitution of deaths to other locations. Furthermore, during the same time period in the surrounding areas observed trends in suicide numbers either increased or remained somewhat stable, suggesting the reduction in deaths at the bridge was not due to wider trends in suicide at the time.

A further three studies in Australia examined the influence of implementing a package of measures (including CCTV, and also fencing, crisis telephones, signage and landscaping works) as part of a large suicide prevention project at one coastal location.^24^,^26^ The latter two studies also mention a sensor-based ‘virtual fence’ installed approximately 3 years after the core package of measures, including the CCTV, which were the focus of these studies (however, the installation of the virtual fences does fall within these study timeframes). None of the studies reported a significant reduction in suicide deaths in the periods after CCTV installation (ie, 2–3 years,^24^ 6 years^25^ or up to 9 years^26^). However, in the most recent study,^26^ exploratory kernel density analysis indicated that, after the project had been implemented, the distribution of the number of suicides occurring at specific areas within the immediate environment may have changed. Specifically, one area within the environment that had been associated with the greatest number of deaths before the measures were implemented (actual n=41, expected n=36) had fewer than expected deaths in the period after (actual n=34, expected n=38), although this change was not significant. There was an increase in deaths at a second location in the same park after the measures were implemented (pre n=9, post n=18), and this was greater than expected (expected values: pre and post n=13). The authors noted that the close proximity of both sites may suggest immediate displacement, and that some people may have been willing to find areas with reduced visibility. However, due to data sparseness they were unable to conduct inferential spatial analysis. Wider trends across the city and broader area were also taken into account by the authors.^26^

Additionally, Lockley *et al*’s^24^ study identified a significant increase in the overall number of police call-outs at the same coastal location over time, but noted that the proportion of episodes where an individual was located ‘over the fence’ was significantly reduced. They suggested that may be likely due to fence improvements but acknowledged that the cameras may have also played a role in aiding earlier intervention. Indeed, as the CCTV system was improved over time, both the number and proportion of successful CCTV assistance requests from police increased (from 9% in 2010 to 40%). This may suggest that the CCTV system supported rescue responses in some cases.

### Studies with systems developed to detect ‘presence’ (eg, motion, intrusion, obstacle)

Across the literature, a range of technologies have been used or proposed to aid the detection of individuals within an environment or crossing a virtual boundary. Such systems may integrate sensors or apply analytics to detect changes within an environment (eg, introduction of body heat, new objects within a region). Unlike non-SST (eg, CCTV cameras) which rely on continuous real-time monitoring, SST may produce automated, technological responses at the site itself (eg, audible deterrents) instead of or in addition to notifying officials (eg, control room staff).

We identified six studies testing the effectiveness of systems designed to detect the ‘presence’ of a person: three focusing on suicide-related outcomes^28 29 34^ and three for trespass more broadly.^33 35 37^ Four of these studies focused on railway environments, specifically; stations, rail-bridges, tunnels and lineside. Two further studies investigated different SSTs installed on bridges with pedestrian access; however, both included data from the same bridge and time period.^28 29^

### Systems which notify for human-led response

One study in South Korea evaluated a system designed primarily to alert control centre staff and was piloted on two river bridges throughout 2013, comprising primarily of an infrared sensor and smart camera with intrusion detection zone.^29^ One of the bridges was reported to have seen a 520% increase in rescues: from 15 in 2012 to 93 in 2013 (including eight on the water). However, due to the cross-over with installation dates of another pilot on this bridge (ie,^28^) it is not possible to attribute any change in rescue numbers to this project.

### Systems which activate lighting

We also identified two studies which included SSTs which activated lighting-based interventions. In one study investigating the installation of a package of measures, including motion-sensitive lighting, signage and platform end barriers, at one railway station, Erlangsen *et al*^34^ reported that no deaths had occurred in the 14 months after installation. The years prior to installation (2012–2018) had seen an average of 1.5 deaths per year, sometimes at secluded parts of the platform. The authors were unable to produce conclusive evidence regarding effectiveness due to the short timeframe, nor would it be possible to attribute effects to any single measure.

In another study examining a variety of different suicide prevention measures,^28^ they present the 2011–2014 descriptive data around the ‘Bridge of Life’ project (deployed at the same bridge in South Korea as the pilot reported by^29^). This system, installed in the latter half of 2012, used motion detection sensors to dynamically illuminate ‘messages of hope’. As previously noted, suicide attempts at the bridge increased from 15 in 2012 to 93 in 2013, and then 184 in 2014. However, in marked contrast, the number of deaths by suicide at the site appeared to remain stable (6 in 2012, 5 in 2013, 5 in 2014). The authors suggest that the increase in attempts may be in part due to the substantial media attention towards the project. Indeed, descriptive statistics summarising events from across five other local bridges suggest attempts remained relatively stable between 2011 and 2013 before almost doubling in 2014.

### Systems which emit audible deterrents

No studies identified to date have examined the effectiveness of using announcements or ‘audible deterrents’ on suicide-related outcomes. However, we identified three reports of initiatives where such a system was tested against trespass-related outcomes, all on the railways. First, de Silva *et al*^37^ reported findings from a system trialled in the USA between 2001 and 2004 that notified a security control room when trespassers were detected on a railway bridge. Staff then activated the loudspeaker to make a scripted announcement requesting trespassers leave the area. There was a reported 60% reduction in trespass at the site between year 1 (46 events) and year 2 (18 events), and then a subsequent somewhat smaller increase in trespass in year 3 (38 events). The authors suggest that this rise may have been due to desensitisation over time in some individuals who trespassed repeatedly without consequence (eg, police intervention). However, they also noted that there had been media coverage in year two about the project and prior fatalities at the location.^37^

Another project tested the effectiveness of an automated audible deterrent installed at two lineside locations on the railways in Finland for approximately 2 months.^35^ At Site A, the installation of the system was associated with a 44% reduction in daily trespass (λ=0.56, 95%CI 0.50 to 0.62). Site B saw an 18% reduction (λ=0.82, 95%CI 0.70 to 0.94). The authors noted that the difference may have occurred because site A had a higher daily number of trains than site B (120 vs 21), which also travelled at a faster speed (up to 120 km/hour vs 50 km/hour). As such, the perceived risk potential of trespassing could have been greater at site A than for site B, which may explain the differences in effect sizes.

Finally, Van Overmeiren described a piloted system which issued audible deterrents when trespassers were detected at a railway tunnel in Belgium.^33^ Descriptive statistics suggest there had been 11 trespass events in the 2 years prior to installation. The year after installation saw two trespass events, and the subsequent year saw none. The installation was also associated with a reduction of 800 delay minutes (−14.8%) for trains in the area from the year prior to installation compared with the first year.

### Studies with systems developed to detect specific ‘actions’

We identified two studies which tested the effectiveness of suicide prevention measures which included SSTs designed to detect specific ‘actions’, both being at bridge locations and intended to alert a rescue team.^30 31^

Using data from a road-bridge in South Korea (2008–2022), Shin *et al*^30^ investigated the effect of installing a Video-based Incident Detection System (VIDS) in 2015 which utilises CCTV cameras and speed sensors to detect vehicles travelling below 30 km/hour, and also the addition of a 2 m high spinning bar rail in late 2017 (previous to these upgrades, the bridge had only a standard 1 m rail and CCTV system). Incident rate ratios (IRR) indicated a significant increase in interventions following the installation of VIDS (IRR=2.40, 95% CI 1.65 to 3.47), but this reduced following the installation of the barrier rails (IRR=0.37, 95% CI 0.25 to 0.57). There was no significant effect of installing VIDS on the number of deaths by suicide (IRR=1.23, 95% CI 0.59 to 2.56), but there was again a reduction in deaths after barrier rails were installed (IRR=0.23, 95% CI 0.07 to 0.71). The author suggests that VIDS may allow atypical behaviours to be identified that could have otherwise been missed, potentially including individuals experiencing different degrees of crisis.

Another study examined a variety of partial restriction measures across four bridges in three countries, including a site where in late 2016, a 1 m upper barrier of five tension wire sensors topped with a spinning rail had been installed atop an existing 1.5 m barrier.^31^ While the five wires had spaces between them, attempts to climb (eg, by pulling a wire by 10 cm or more) or cut the wire sensors would lead the SST to alert a rescue team. Using data from 2013 to 2020, the authors reported a significant decrease in the number of suicides in the period after installation (IRR=0.37, 95% CI 0.25 to 0.54). Shin *et al*^31^ suggested that this may be because the wires and spinning rail make access more difficult. As intervention-related outcomes were not reported, it is difficult to ascertain the extent to which the sensors may have also played a role in the positive outcome (eg, automating notifications of climbing attempts to rescuers).

### Quality appraisal

The quality appraisals of the included studies are reported in online supplemental appendix C. Studies achieved between 0% and 60% of the MMAT quality criteria for quantitative non-randomised studies. It is important to note, however, that study ratings were affected due to MMAT Q.3.1 which asks about the representativeness of participants, which in the case of these studies was not appropriate. Moreover, five of the studies^24^,^2629 37^ identified changes to the way in which the systems operated (eg, periods where the SST was not operational, upgrades to the system), and it was unclear whether such changes had occurred during the data collection periods for the other studies. Overlap with the installation of other mitigating initiatives (either as part of the same study or reported elsewhere) meant that confounders could not be accounted for in several of the studies.^23^,^2628 29 34^ Also, two studies did not provide sufficient information to determine the appropriateness of measurements or completeness of outcome data.^28 29^

## Discussion

In this systematic review, we identified 15 studies which looked at whether the presence or installation of surveillance technologies (often alongside other measures) influenced rates of suicide or related events at public locations. Overall, the evidence provided by these studies is mixed. Increases in interventions or rescues were identified in two studies after SSTs were installed on bridges to detect vehicles slowing down^30^ and people leaning over railings.^29^ Elsewhere, a package of measures installed at a coastal location (including a 1.3 m barrier and a CCTV system) was also associated with an overall increase in police call outs, a decreasing proportion of which involved ‘over the fence’ call outs.^24^ Given that surveillance technologies are often categorised as a measure to help increase opportunity for human intervention, these associations may be somewhat expected. Despite the associated increase in rescues/interventions in these studies, however, only one reported a significant reduction in deaths. This was not associated with the installation of the SST, however, but the subsequent addition of a 2 m barrier.^30^ Similarly, while Kõlves *et al*^23^ suggest that the decline in suicides at one bridge site in Australia began following the installation of cameras and crisis phones, there was a much clearer reduction in deaths following the installation of 3 m barriers at the site 3 years later.

Across the review there was only one study identified where the introduction of measures, including an SST, was statistically associated with a reduction in deaths.^31^ In this initiative, components of the SST (eg, tension wire sensors) were integrated into a physical measure installed at one bridge that partially restricted access to means. Unlike the physical mitigations deployed in the aforementioned studies,^23 30^ the installation of these sensors (which were also topped with a spinning rail) would not prevent an individual climbing through them, but would alert officials of any attempts to climb through or cut them. From this perspective, such a mitigation may help to ‘restrict access to means of suicide’ and also ‘increase opportunity for human intervention’. However, as the study findings were limited to deaths only, it is not possible to confirm the latter. While these data may not always be collected or straightforward to obtain, there may be benefits in reporting on alternative outcomes (eg, interventions) in studies where surveillance technologies are part of the intervention. Further work is also needed to understand whether the introduction of tension wire sensor-based SSTs may lead to unintended consequences (eg, displacement or substitution effects). Three studies compared the characteristics of multiple locations across transportation networks, and as such, did not focus exclusively on ‘high-frequency’ locations for suicide or trespass.^27 32 36^ In two of these studies, the presence of surveillance technology at subway stations (after controlling for other environmental factors) was not associated with significant differences in rates of suicide attempts or deaths by suicides compared with stations without surveillance technology.^32 36^ However, another study found that, after controlling for other factors, the number of surveillance ‘units’ identified at railway stations and station car parks within a neighbourhood was negatively associated with rail suicide rates.^27^ Accounting for the (perceived) coverage of surveillance (rather than simple presence of a camera) may be useful in this context. Indeed, Uittenbogaard and Ceccato^36^ found significant associations between suicide risk at station and factors that may impact perceived visibility, such as the presence of over 20 passengers or walls dividing platforms. Moreover, Torok *et al*^26^ suggested signs of immediate displacement within a cliff site environment may reflect a willingness to seek out areas with reduced visibility (eg, not covered by cameras). This aligns with previous work that suggests some people may actively avoid surveillance technology when looking to access means.^18 39^ Further research is therefore needed to better understand whether perceptions of surveillance and surveillance coverage have an overall impact on suicidal behaviours at high-risk locations.

While many of the studies featuring surveillance technologies were designed to initiate or aid human-led interventions, several initiated technology-led responses. Studies which included SSTs designed to initiate motion-activated or dynamic lighting offered no conclusive evidence of preventing suicides. No studies of the impact of audible deterrents (either automated or issued remotely from a control room) on suicide outcomes were found in the search. However, three studies examined the effect on trespass.^33 35 37^ Although the findings from these studies suggest that SSTs that issue audible deterrents may be helpful for reducing trespass on the railways, their application for suicide prevention is unclear. While further research is required, there is some initial evidence to suggest that perceived risks and consequences may have played some role in determining the effectiveness of audible deterrents at reducing trespass. Indeed, de Silva *et al*^37^ found that the effectiveness of an SST installed at one rail-bridge location waned over time, potentially due to individuals becoming desensitised to the announcement and awareness that a human-led response was unlikely to arrive. Additionally, Kallberg and Silla^35^ found that the system was more effective at reducing trespass when deployed at a site with a higher frequency of trains that also travelled faster (ie, a higher-risk site). More broadly, other studies have found that a combination of education on rail safety and threat of punishment may help to prevent unsafe behaviours at level crossings.^40 41^ Yet, Barić *et al*^40^ found unsafe behaviours were not influenced by installing CCTV alone. As such, the findings that audible deterrents may help prevent trespass may not necessarily translate to suicide prevention. Although some suicide attempts may involve a degree of trespass, the intended outcome is arguably very different from someone who, for example, intends to pass through the environment as a shortcut. Given that train frequency and speed have previously been associated with risk of rail suicide at a location,^27 39 42^ perceived risk and consequences may be less likely to reliably act as preventive factors in the context of suicide (vs general trespass).

There were several limitations around the evidence identified in this review. A key limitation is that we only included evidence published in English. As identified in this review, much of the work on SSTs in suicide prevention takes place in countries where English is not the official language. Future research in this area may therefore benefit from international collaboration. Second, while fifteen studies were identified, three examined one suicide prevention project at different points in time,^24^,^26^ and potentially three studies looked at different projects installed on the same bridge.^28 29 31^ Our knowledge about surveillance technologies is therefore limited to a small number of sites. Moreover, we found SSTs were frequently installed alongside a package of other measures. This means their contribution is often difficult to gauge. In addition, descriptions of SST capabilities and surrounding processes are often limited or absent. Other challenges associated with working with real-world data, such as data availability (eg, of additional outcomes) and the relative rarity of an event such as suicide (eg, meaning that certain inferential tests are not always feasible) are another limitation of the evidence in this area.

### Implications for practice, policy and future research

Findings from this review highlight critical gaps in the current evidence. While our initial searches and previous work identified a broader and often more advanced range of surveillance technologies being proposed and deployed for suicide prevention,^18^ the mechanisms for detection used by surveillance technologies in the studies identified here were relatively limited in comparison (eg, motion detection), or non-existent (eg, CCTV). Whether the findings would therefore extend to other detection approaches is unclear. It does, however, raise important questions around the ethics of using SST for suicide prevention in public spaces without evidence not only supporting their effectiveness but also cost-effectiveness. There are also complex legal and ethical issues surrounding the use of SSTs that deserve further attention (these have begun to be explored in more detail elsewhere^18 43 44^). Efforts to understand what people with lived experience feel about these technologies are vital for ensuring their equitable and acceptable use.^44^

The evidence around whether SSTs may have a direct impact on preventing suicides is currently limited. Well-designed natural experimental studies comparing trends in larger samples of matched intervention and control sites are needed. Moreover, only one study found a significant reduction in suicides following the installation of an SST, and this was notably the one that also included partially restricted access to means via the use of tension-wire sensors.^31^ Understanding how SSTs may be integrated with physical mitigations to aid suicide prevention efforts may therefore be a useful avenue for future research.

While there was also some evidence to suggest that fully automated audible deterrents may be effective for reducing trespass rates,^33 35^ no studies explored whether they would also help to prevent suicides. An important consideration is whether any perceived risks that may deter a trespasser from taking a shortcut (eg, serious injury) after hearing an automated warning are likely to apply to individuals motivated to make a suicide attempt. Indeed, previous research has highlighted a potential risk that some individuals may move to act more quickly during a suicide attempt if they perceive an imminent intervention.^25 45^ Others have raised concerns that SSTs could draw attention to a site,^18^ and this is something one study also identified as a factor potentially explaining why there was a notable increase in the number of suicide attempts following the implantation of a dynamic lighting intervention at one bridge.^28^ Given these potential risks and the lack of empirical support, a cautious approach to fully automated SSTs (eg, audible deterrents) is warranted, with further research required to establish their efficacy and safe use in suicide prevention context.

Our findings also suggest that the way in which surveillance technology feeds into wider processes surrounding suicide prevention is an important consideration for practitioners and researchers. In many of the studies identified, surveillance technologies were instead part of the intervention process supporting humans to identify events and initiate a response. From this perspective, SSTs are only valuable for suicide prevention if the processes in place to respond are sufficient. Yet, our understanding of how SSTs may indirectly help to prevent suicides in this way remains limited. The installation of SSTs developed to send notifications to staff was associated with an increase in interventions in two studies.^29 30^ However, most other studies did not examine this outcome. Information about the monitoring of systems and any response mechanisms was also limited across studies where a human-led response would be required. Future research in this area should consider what happens following an alert, as technology is only one part of an intervention in this context (eg, processes, response times). An SST may be able to detect a person in crisis, for example, but the time it then takes a rescue response to be organised and then arrive at a site may inform whether the technology can actually help to prevent suicides in the real world. Evaluating SSTs against multiple outcomes (eg, other suicidal behaviours, average response times) may therefore be valuable for understanding both their effectiveness at preventing suicides and the implications for their use for those working on the ground.

## Supplementary material

10.1136/bmjph-2025-004286 online supplemental file 1

## Data Availability

All data relevant to the study are included in the article or uploaded as supplementary information.
